# 2392: Detecting cardiometabolic disease through breath analysis: A metabolomic approach

**DOI:** 10.1017/cts.2017.229

**Published:** 2018-05-10

**Authors:** Ahsan Choudary, Andrew C. Bishop, Biswapriya Misra, Mark Libardoni, Kenneth Lange, John Bernal, Mark Nijland, Cun Li, Peter W. Nathanielsz, Michael Olivier, Laura A. Cox

**Affiliations:** Texas Biomedical Research Institute & Southwest National Primate Research Center, San Antonio, TX, USA

## Abstract

OBJECTIVES/SPECIFIC AIMS: The purpose of this study is to use the baboon as a novel animal model for breath research and to identify and characterize baboon breath metabolites that reflect cardiometabolic function to inform us in the development of a noninvasive, cost-effective, and repeatable point-of-care diagnostic breath test. METHODS/STUDY POPULATION: Blood and urine was collected from control and IUGR at the approximate age of 3.5 years. Both groups were then placed on a high fat, high sugar, high salt diet for 7 weeks, after which blood, urine, and breath were collected. The breath samples were then subjected to comprehensive, 2-dimensional gas chromatography coupled with time-of-flight mass spectrometry. Using ChromaTOF software, breath VOCs were identified with at least an 80% spectral match against the National Institute of Standards and Technology (NIST) chemical reference library. The raw data were then statistically analyzed using MetaboAnalyst. We then interrogated multiple online databases to characterize and identify the role of VOCs that were present in both control and IUGR groups. RESULTS/ANTICIPATED RESULTS: Preliminary analyses of the breath VOCs indicate differences in expression between sexes and in control Versus IUGR groups. These results indicate unique “breath signatures.” Further analysis of the breath VOCs reveals the presence of metabolites that are involved in β-oxidation and oxidative stress pathways. DISCUSSION/SIGNIFICANCE OF IMPACT: This breath study, a first of its kind, will develop the baboon as a superior animal model for breath biomarker research. Our observed unique “breath signatures” indicate changes in lipid metabolism and oxidative stress pathways, which we hypothesize are the early metabolic changes at the cellular level that are not yet reflected in clinical lab measures. Future directions include analyzing breath VOCs that did not meet 80% spectral match, validation using SPME technology and commercial standards, and initiating a human pilot study in clinically obese, at-risk children in collaboration with physicians at the Children’s Hospital of San Antonio to develop a noninvasive, cost-effective, rapid, and repeatable point-of-care diagnostic breath test.

